# The Patients are Thriving: Further Evidence of Improved Outcomes for Women Surgeons

**DOI:** 10.1097/AS9.0000000000000504

**Published:** 2024-10-11

**Authors:** Juliet Emamaullee, Kambiz Etesami

**Affiliations:** * Division of Abdominal Organ Transplantation and Hepatobiliary Surgery, Department of Surgery, Keck School of Medicine, University of Southern California, Los Angeles, CA.

With an expanding emphasis on value-based care delivery, quality metrics have become center stage in healthcare. For surgeons, factors that contribute to patient outcomes have been examined across the spectrum of surgical care: preoperative, perioperative, intraoperative, postoperative, and postdischarge. One of the most intriguing variables that has been associated with improved outcomes in surgical patients has been surgeon gender. Indeed, even when controlling for many variables, including procedure type, anesthetist, and duration of surgery, large registry studies have shown that care provided by female surgeons has been linked to less postoperative complications and lower costs of care.^1,2^ Upon further investigation, sex discordance between surgeon and patient only partially accounted for these observations, as both male and female patients had improved outcomes (death, reoperation, or complications within 30 days) when operated on by female surgeons, while female patients being treated by male surgeons had worse outcomes.^3^ The underlying cause for these observations remains to be determined and has generated much debate.

In this issue, Heybati et al^4^ examine the impact of surgeon gender on a postdischarge quality metric, ‘days alive and at home’ (DAH) following surgery. This validated quality measure that includes length of stay, readmissions, and discharge to a nonhome location was used as an indicator of postdischarge care delivery for 25 common surgical procedures. Using a similar registry dataset from Ontario, Canada that was used for prior surgeon gender-based studies from this group, they examine DAH at 30, 90, and 365 days postoperatively.^1–3^ They observed that DAH was higher for patients operated on by female surgeons at all time points, although the differences were subtle (25.6 *vs* 23.9 days at 30 days, 83.7 *vs* 80.5 days at 90 days, and 351.7 *vs* 342.1 days at 365 days for female *vs* male surgeons with *P* < 0.0001 at each interval). Next, they adjusted for several co-variables including patient demographics (age, sex, comorbidities, income quintile, and rurality) and surgeon/anesthetist characteristics (age, years in practice, surgical specialty, and annual case volume), duration of surgery, and hospital characteristics, and still observed longer DAH for female surgeons at each time interval (*P* < 0.001 *vs* male surgeons). Taken together, these data further support the concept that receiving care from female surgeons results in less complications, lower costs, and ultimately improved postdischarge quality of life, at least as extrapolated from the DAH metric.

Overall, these findings highlight the continued need to explore why female surgeons achieve higher-quality outcomes across different phases of surgical care. In contrast to the evaluation of quality metrics by surgeon gender favoring women surgeons, much of the literature examining the surgical profession reveals that female surgeons experience many challenges. When compared to their male counterparts, female surgical residents have higher attrition rates, lower case volumes, less operative autonomy, receive lower evaluation scores, and are less likely to receive awards in residency.^5–8^ As female surgeons progress into faculty positions, they often encounter misogynistic work environments, experience higher rates of burnout, and are less likely to be on the podium at conferences or serve in leadership roles for surgical societies when compared to male surgeons.^9–12^ Further, despite the recognized increased value of having a female surgeon reported in this issue and in this group’s prior work, analysis of the same Ontario registry data has identified a significant pay gap for female surgeons, who earned 24% less per hour of operating, partially as a result of performing ‘less lucrative’ procedures in a fee-for-service reimbursement model.^13^ Collectively, these studies suggest that despite the barriers female surgeons experience in their training and professional careers, they continue to provide high-quality care to their patients.

In the current value-based healthcare system, data like those reported by Heybati et al^4^ should be leveraged to ensure that female surgeons achieve pay parity and are supported in their work environments to enable professional growth and minimize the negative impact of workplace inequities and physician burnout. One might speculate that barriers in training, combined with higher rates of attrition, could inadvertently select for more resilient and higher-performing women surgeons pursuing careers in surgery. As the proportion of female surgeons continues to expand, future research should attempt to dissect out factors contributing to superior outcomes for female surgeons, such that these can be addressed to ensure the best outcomes for our patients, independent of surgeon gender.

## Acknowledgment

J.E. wrote the manuscript. K.E. provided key guidance and oversight. Both J.E. and K.E. edited the manuscript prior to submission.
